# Efficacy of tocilizumab for hospitalized patients with COVID-19 pneumonia and high IL-6 levels: A randomized controlled trial

**DOI:** 10.1007/s15010-025-02506-y

**Published:** 2025-04-15

**Authors:** Júlia Sellarès-Nadal, Juan Espinosa-Pereiro, Joaquín Burgos, Vicenç Falcó, Alfredo Guillén-Del-Castillo, Salvador Augustin, Juan Bañares-Sánchez, Alba Prio-Ruatg, Ferran Martínez-Valle, Cristina Kirkegaard-Biosca, Adrián Sánchez-Montalvá

**Affiliations:** 1 Department of Medicine, Universitat Autònoma de Barcelona, Bellaterra, Spain; 2 Infectious Diseases Department, Vall d’Hebron Barcelona Hospital Campus, Vall d’Hebron Hospital Universitari, Passeig Vall d’Hebrón 119-129, 08035 Barcelona, Spain; 3 Malalties Infeccioses Vall d’Hebron Institut de Recerca (VHIR), Vall d’Hebron Barcelona Hospital Campus, Vall d’Hebron Hospital Universitari, Barcelona, Spain; 4 Infectious Diseases Department, International Health Unit Vall d’Hebron-Drassanes, Vall d’Hebron University Hospital, PROSICS Barcelona, Barcelona, Spain; 5 Centro de Investigación Biomédica en Red de Enfermedades Infecciosas (CIBERINFEC), Instituto de Salud Carlos III, Madrid, Spain; 6 Internal Medicine Department, Vall d’Hebron Barcelona Hospital Campus, Vall d’Hebron Hospital Universitari, Barcelona, Spain; 7 Hepatology Department, Vall d’Hebron Barcelona Hospital Campus, Vall d’Hebron Hospital Universitari, Barcelona, Spain

**Keywords:** Tocilizumab, Interleukin-6, COVID-19, Viral pneumonia, Acute respiratory distress syndrome, Randomized controlled trial

## Abstract

**Background:**

The objective of this clinical trial is to evaluate the efficacy and safety of IL-6 driven personalized treatment strategy with tocilizumab in patients with severe COVID-19 pneumonia.

**Trial design:**

Randomized, controlled, open-label, single-center trial of a tocilizumab treatment strategy in adult patients hospitalized with severe COVID-19 pneumonia and IL-6 serum levels > 40 pg/mL.

**Methods:**

Patients were randomized 1:1 to receive standard of care (SOC) or SOC plus one dose of tocilizumab. The primary outcome was death or need for invasive mechanical ventilation (IMV) within 28 days after randomization. Secondary outcomes included ICU admission, days on IMV and hospital stay. A meta-analysis of clinical trials to evaluate the effect of tocilizumab on mortality and need of IMV in patients with COVID-19 pneumonia was performed.

**Results:**

Sixty-two patients were included: 30 in the SOC arm and 32 in the standard-treatment plus tocilizumab arm. The primary outcome occurred in 12.9% in the tocilizumab arm and 32.3% in the SOC arm(p = 0.068). There was a trend towards fewer days on IMV (7.5 vs 19.5 days, p = 0.073) and a shorter hospital stay (4 vs 8 days, p = 0.134) in the tocilizumab group. No serious adverse events were reported. The meta-analysis revealed a RR for death or IMV of 0.83 (95% CI: 0.77–0.89) in patients receiving tocilizumab, compared to patients receiving SOC.

**Conclusion:**

Tocilizumab could be effective to prevent death or IMV in patients with severe COVID-19 pneumonia and high IL-6 serum levels. Safety profile of tocilizumab does not arise major concern in patients with severe COVID19.

**Supplementary Information:**

The online version contains supplementary material available at 10.1007/s15010-025-02506-y.

## Introduction

COVID-19-related pneumonia can trigger a hyperinflammatory response, marked by elevated levels of cytokines, such as interleukin-6 (IL-6). [1, 2]. This so-called cytokine storm has been associated with worse clinical outcomes, including acute respiratory distress syndrome (ARDS) and increased mortality [3, 4]. Tocilizumab, an IL-6 receptor antagonist, has demonstrated efficacy in cytokine release syndromes such as CAR T-cell-induced cytokine storm [5], leading to its investigation as a potential therapeutic option in patients with severe COVID-19 pneumonia.

Both the Infectious Diseases Society of America (IDSA) and the European Society of Clinical Microbiology and Infectious Diseases (ESCMID) recommend tocilizumab for hospitalized adults with severe COVID-19 [6, 7]. This recommendation is based on the results of several observational studies and clinical trials (including large platform trials) suggesting a benefit of tocilizumab in people with severe COVID-19 pneumonia and associated ARDS [8, 9]. Other clinical trials, however, have shown contradictory results [10, 11]. Some authors hypothesize that these differences may be influenced by standard care, especially regarding the use of corticosteroids, ethnic differences in the recruited subjects, the timing and dose of tocilizumab and whether this therapy should be guided by cytokine levels. This approach could potentially enhance the selection of patients with the highest likelihood of responding to tocilizumab [12].

Therefore, there is a need to personalize the treatment with tocilizumab and identify patient subgroups most likely to benefit from tocilizumab. IL-6 serum levels have been associated with high risk of poor prognosis in patients with severe COVID-19 so, guiding tocilizumab treatment according to the levels of IL-6 could be a strategy to maximize outcomes, and optimize the proper use of a limited and expensive resource.

The objective of this study was to assess the efficacy and safety of an IL-6 serum level driven strategy for the administration of tocilizumab in adult patients hospitalized with severe COVID-19 pneumonia. Unlike previous trials, we specifically targeted patients with IL-6 serum levels > 40 pg/mL, a threshold associated with poor clinical outcomes. This study aims to refine the therapeutic role of tocilizumab and optimize its cost-effectiveness by selecting patients with COVID-19 pneumonia and increased IL-6.

## Material and methods

### Trial design and participants

We performed a randomized, controlled, open-label, single-center trial of an IL-6 driven tocilizumab treatment strategy in adult patients hospitalized with severe COVID-19 pneumonia.

Eligible participants were recruited between April 2020 and June 2022. We included adults in the age range from 18 to 80 years old hospitalized with severe SARS-CoV-2 pneumonia with IL-6 serum levels ≥ 40 pg/ml (normal values ranging from 0 to 4.3 pg/mL). All participants had a positive PCR or EU-recommended antigen test for SARS-CoV-2 in a nasopharyngeal swab. The exclusion criteria were liver enzyme cytolysis elevation greater than 5 times the upper limit of normal, neutropenia fewer than 0.5 × 10E9/L, thrombocytopenia with less than 50 × 10E9/L platelets, sepsis or pneumonia caused by other pathogens, pregnancy and other conditions that contraindicate tocilizumab administration.

### Definitions

We defined a severe SARS-CoV2 pneumonia as a newly visualized infiltrate in the chest radiography and at least one of the following severity criteria: (1) peripheral arterial oxygen saturation breathing room air ≤ 94% measured by pulse oximetry, (2) Partial pressure of oxygen / fraction of inspired oxygen (Pa:FiO2) ≤ 300, (3) peripheral arterial oxygen saturation measured by pulse oximetry / fraction of inspired oxygen (Sa:FiO2) ≤ 350.

Intensive care unit (ICU) admission was defined as participants requiring intensive monitoring and care, including high-flow nasal cannula, invasive or non-invasive mechanical ventilation (IMV and NIMV, respectively) and vasoactive drugs. This classification was applied, regardless of their physical location within the hospital.

### Interventions

Participants were assigned in a 1:1 ratio to receive standard of care (SOC) treatment according to national guidelines at the time of inclusion or to receive SOC treatment plus a single dose of weight-based intravenous tocilizumab: 400 mg for participants weighing < 75 kg and 600 mg for those weighing ≥ 75 kg 600 mg. The SOC evolved during the pandemic as new evidence and new compounds were available. This included antiviral therapies, steroid therapy, and other immune modulating therapies (i.e., azithromycin, tocilizumab). According to this, the protocol allowed that participants allocated to the control arm could receive tocilizumab if they experienced clinical deterioration after the randomization. The participants were followed for 28 days. Follow-up visits included vital signs, clinical information (symptoms, physical examination, and need of supplementary oxygen), blood-sample results, radiological findings, safety information, treatment information and main study outcomes. Adverse events (AE) were recorded and classified according to the MedDRA system organ-class classification and the CTAE terminology criteria for adverse events [13, 14].

### Outcomes

The primary outcome was a composite of death for any reason or the need of IMV during the follow up (28 days) The secondary outcomes were days of IMV, days of non-invasive mechanical ventilation of high-flow nasal cannula, admission to ICU and hospital stay length.

### Sample size

We conducted the sample size calculation using estimates based on the available literature in September 2020 [11, 15, 16]. The primary outcome was the proportion of patients who die or require mechanical ventilation by day 28, which was estimated in 37% for the control group and 10% for the group receiving tocilizumab. Assuming an alpha error of 0.05 and a beta error of 0.20, with no loss to follow-up and a 1:1 randomization, the sample size calculated was 41 participants per arm.

### Statistical analysis

Data was captured on the electronic health record of the participants and then transferred to a RedCap database. We performed a descriptive analysis of basal characteristics of the study population. Categorical variables were expressed as total numbers and percentages and numerical data as mean and standard deviation (SD) if they followed normal distribution and as median and interquartile range (IQR) otherwise.

**Fig. 1:**
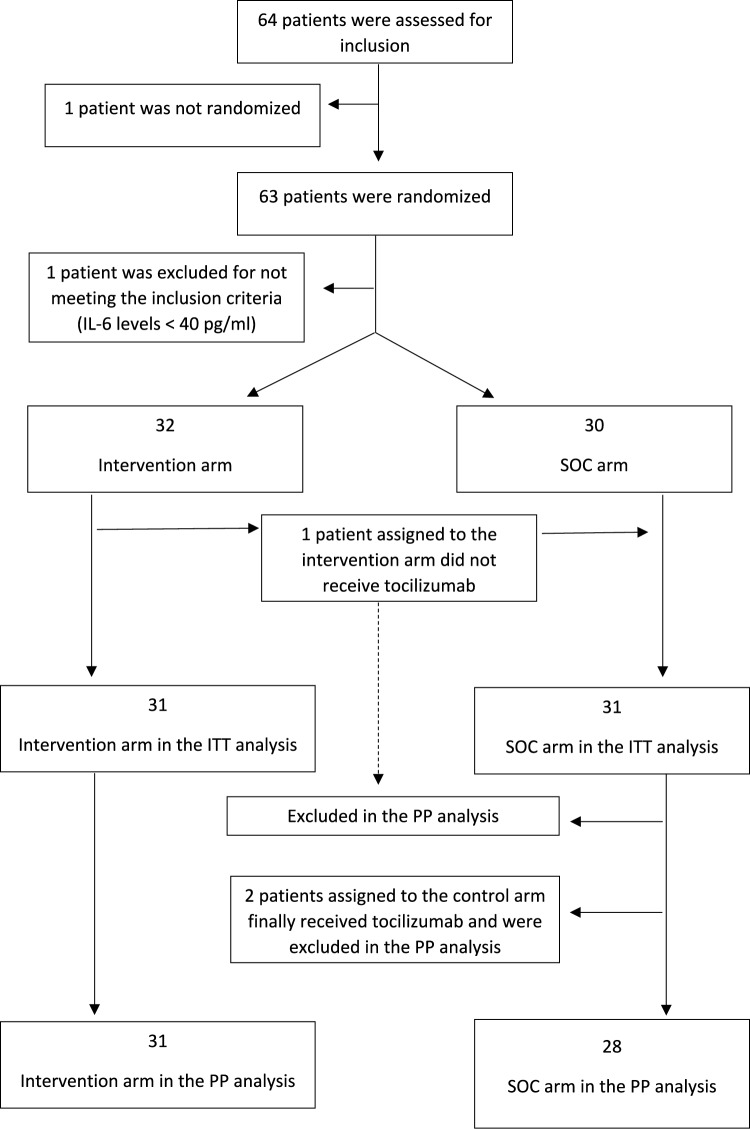
Patients flowchart

In the intention-to-treat (ITT) analysis, patients who received tocilizumab despite being in the control arm were considered failures, while one patient that did not receive tocilizumab despite being in the intervention arm was reassigned to the control group. The per-protocol (PP) analysis exclusively considered patients strictly adhering to the protocol requirements (Fig. 1). A modified intention-to-treat (mITTt) and modified per-protocol (mPP) analyses were performed and are reported in the supplementary material. The mITT reassigned the two patients that received tocilizumab despite being in the control group to the intervention group. The mPP analysis considered these two participants as failures in the control group and excluded the patient that did not receive tocilizumab despite being assigned to the intervention group.

We conducted a survival analysis of the primary outcome using the Kaplan–Meier method and assessed between-group differences in survival using a log-rank test.

The safety analysis cohort consisted in all randomized participants.

### Literature review and meta-analysis

Finally, we performed a systematic research and meta-analysis of studies evaluating the effect of tocilizumab in hospitalized patients with COVID-19 pneumonia. We systematically searched PubMed up to June 2024 in order to identify relevant studies. The search term can be found in the supplementary material. We included clinical trials using mortality or the need of IMV as primary outcome. We did not perform a grey literature search. We performed a risk of bias assessment adapting the RoB-2 tool with the following domains: confounding factors, selection and intervention classification, missing data, outcome measurement and result reporting [17]. After data extraction the results of this trial were pooled with those obtained from the review. The treatment effect was summarized as risk ratio (RR) using the Mantel–Haenszel method and then fitting a random-effects model with a DerSimonial-Laird variance estimation. We assessed inter-study heterogeneity with a restricted maximum likelihood model and reported it as the I^2^ and tau statistics.

The analysis of the trial results was performed using SPSS software for statistical analysis (IBM SPSS Statistics for Windows, Version 20.0. Armonk, NY; IBM Corp.), and the meta-analysis using R software (Vienna, version 4.1.2) and the *meta* package.

### Ethical considerations

The study protocol was designed following the Declaration of Helsinky and Good Clinical Practice guidelines. The EudraCT registration number was 2020-001437-12. The hospital ethical committee and the Spanish Drug Agency (AEMPS) approved the clinical trial. All participants provided written informed consent.

## Results

### Participants

Sixty-four patients were evaluated for inclusion between April 2020 and August 2022. One participant signed the informed consent, but was not randomized, and another participant was excluded for not meeting the inclusion criteria. Finally, 62 subjects were included: 30 in the SOC arm and 32 to the tocilizumab arm. Recruitment was stopped before reaching the target sample size due to project due date, funding constrains and low recruitment rate.

One participant did not receive tocilizumab despite being allocated to the intervention arm. Two participants assigned to the control arm received tocilizumab during the follow-up and therefore were considered to have a poor outcome in the ITT analysis (Fig. 1).

The median age was 60.5 years (IQR 52–70) and 42% were women. Sixty per cent of the participants received oxygen through Ventouri mask at inclusion, with a median fraction of supplementary oxygen of 28% (IQR 26–31). The median IL-6 serum level at inclusion was 76.4 (IQR 53.7–103.8). The median time from symptom onset to inclusion was 9 days (IQR 7–12). Table 1 shows baseline characteristics of the participants, demographic data, past medical history and clinical situation at inclusion did not differ between groups. Other analytical parameters are depicted in the supplementary material (Table S1).

**Table 1:** Baseline sociodemographic and clinical characteristics in both groups

Characteristics	SOC n = 31 (%)	Tocilizumab n = 31 (%)
Basal characteristics		
Median age in years (IQR)	63 (53–72)	57 (47–69)
Female sex n (%)	10 (32.3)	16 (51.6)
Body Mass Index		
BMI < 30 kg/m2	23 (74.2)	21 (67.7)
BMI 30–39.9 kg/m2	8 (25.8)	8 (25.8)
BMI > = 40 kg/m2	0	1 (3.2)
Unknown	0	1 (3.2)
Alcohol (> 60 gr/day)	4 (12.9)	3 (9.7)
Smoker	0	2 (6.5)
Cognitive impairment	0	1 (3.2)
ECOG		
ECOG 0	28 (90.3)	28 (90.3)
ECOG 1	2 (6.5)	2 (6.5)
ECOG 2	0	1 (3.2)
Unknown	1 (3.2)	
Underlying conditions		
Diabetes mellitus	8 (25.8)	9 (29)
Any immunosuppressive condition	1 (3.2)	3 (9.7)
Solid neoplasm history	2 (6.5)	1 (3.2)
Arterial hypertension	18 (58.1)	10 (32.3)
Heart failure	1 (3.2)	0
Ischemic cardiopathy	0	2 (6.5)
Atrial fibrillation	1 (3.2)	0
Pneumopathy	6 (19.4)	5 (16.1)
Asthma	1 (3.2)	0
COPD	2 (6.5)	1 (3.2)
Bronchial hyperreactivity	0	2 (6.5)
Other	3 (9.7)	2 (6.5)
Non-viral chronic hepatopathy	3 (9.7)	1 (3.2)
CNS disease	3 (9.7)	4 (12.9)
Charlson comorbidity index		
CCI 1–2	16 (51.6)	20 (64.5)
CCI 3–4	11 (35.5)	8 (25.8)
CCI > = 5	4 (12.9)	3 (9.7)
Initial symptoms		
Fever	23 (74.2)	28 (90.3)
Cough	24 (77.4)	25 (80.6)
Dyspnea	13 (41.9)	21 (67.7)
Myalgia/arthralgia	12 (38.7)	13 (41.9)
Diarrhea	4 (12.9)	15 (48.4)
Asymptomatic	0	0
Median days from onset of symptoms until inclusion [IQR]	8 [6–11]	9 [8–12]
Clinical basal situation		
Supplementary oxygen or ventilation		
Nasal cannula	11 (35.5)	12 (38.7)
Ventouri mask	19 (61.3)	18 (58.1)
Reservoir mask	1 (3.2)	0
High-flow cannula	0	1 (3.2)
Blood sample results		
Median IL-6 [IQR]	76.6 [52.3–98.5]	76.2 [53.8–118.8]
Treatment		
Corticosteroids^1^	28 (90.3)	24 (77.4)
Cumulative dose of corticosteroids in mg [IQR]^2^	375 [375–375]	375 [375–375]
Hydroxychloroquine	3 (9.7)	9 (29)
Lopinavir/ritonvavir	3 (9.7)	7 (22.6)
Remdesivir	3 (9.7)	1 (3.2)
Antimicrobial therapy^3^	7 (22.6)	10 (32.3)
SARS-CoV 2 vaccine		
Not vaccinated	19 (61.3)	22 (71)
1 dose	6 (19.4)	5 (16.1)
2 doses	5 (16.1)	3 (9.7)
3 doses	1 (3.2)	1 (3.2)

Data presented as n (%) and mean (SD) otherwise specified

*SOC* Standard of care, *ECOG* ECOG performance status scale, *COPD* Chronic Obstructive Pulmonary Disease, *CCI* Charlson Comorbidity Index, *CNS* Central nervous System, *IL-6* Interleukin 6

^1^Corticosteroids: Dexamethasone, Prednisone, Methylprednisolone

^2^Cumulative dose in mg of prednisone equivalent

^3^Antibiotics: Ceftriaxone, Amoxicillin-clavulanic acid, Piperacillin-tazobactam

Regarding concomitant medications, 52 participants (83.9%) received corticosteroids with a median cumulative dose of 375 mg of prednisone. Moreover, 12 (19.4%) participants were prescribed hydroxychloroquine, 4 (6.5%) remdesivir, 10 (16.1%) lopinavir/ritonavir and 17 (27.4%) antimicrobial therapy, with no differences between groups (Table 1). There were no differences in terms of vaccination status between groups.

### Efficacy outcomes

The ITT analysis revealed that the death or need for IMV percentage by day 28 was 12.9% in the participants in the tocilizumab group and 32.3% in the participants in the SOC group, although the comparison did not reach statistical significance (p = 0.068) (Table 2). Results from the PP analysis are depicted in Table 2. Additionally, the modified ITT and modified PP are shown in the supplementary material (Table S2).

**Table 2:** Primary and secondary outcomes

Intention-to-treat (ITT) analysis	SOC n = 31	Tocilizumab n = 31	P value
Death or IMV by day 28	10 (32.3)	4 (12.9)	0.068
Death	2 (6.5)	1 (3.2)	0.549
IMV	8 (25.8)	4 (12.9)	0.199
Secondary outcomes			
ICU (IMV, NIVM, HFNC) by day 28	14 (38.7)	13 (41.9)	0.799
Death or hospitalized at the ICU at day 28	7 (22)	2 (6.5)	0.07
Median days (IQR) under*			
Invasive mechanical ventilation (n = 12)	19.5 (10.3–36.8)	7.5 (2.3–12.8)	0.073
High-flow nasal cannula or NIMV (n = 24)	3.5 (2–6.8)	6 (3–6.8)	0.319
Median length of hospital stay since the study inclusion (IQR)	8 (6–20)	4 (4–13)	0.134

Per-protocol (PP) analysis	SOC n = 28	Tocilizumab n = 31	P value
Death or IMV by day 28	7 (25)	4 (12.9)	0.234
Death	1 (3.6)	1 (3.2)	0.933
IMV	7 (25)	4 (12.9)	0.234
Secondary outcomes			
ICU (IMV, NIVM, HFNC) by day 28	12 (42.9)	13 (41.9)	0.938
Death or hospitalized at the ICU at day 28	4 (14.3)	2 (6.5)	0.320
Median days (IQR) under*			
Invasive mechanical ventilation (n = 11)	14 (10–27)	7.5 (2.25–12.8)	0.109
High-flow nasal cannula or NIMV (n = 21)	3 (2–5)	6 (3–6.8)	0.129
Length of hospital stay since the study inclusion, median, (IQR)	8 (5.3–16)	7 (4–13)	0.253

*ICU* Intensive care unit, *IMV* Invasive mechanical ventilation, *NIVM* Non-invasive mechanical ventilation, *HFNC* High flow nasal cannulaQuantitative data is presented as total number as percentage

^*^Medians presented only among patients receiving IMB or HFNC and NIMV

Regarding secondary outcomes, the rate of participants fulfilling ICU admission criteria during the follow-up were similar between groups (tocilizumab 41.9% vs SOC 38.7%, p = 0.799). Despite this, there was a trend toward fewer rates of participants that were dead or remained hospitalized at the ICU at the end of study follow-up in participants in the tocilizumab group (tocilizumab 6.5% vs SOC 22%, p = 0.07). Moreover, there was a non-significant trend towards the length of stay under IMV (tocilizumab 7.5 vs SOC 19.5 days, p = 0.073) and shorter hospital stay in the tocilizumab group (tocilizumab 4 vs SOC 8 days, p = 0.134) (Table 2). The PP analysis brought similar results in the primary and secondary outcomes (Table 2).

**Fig. 2:**
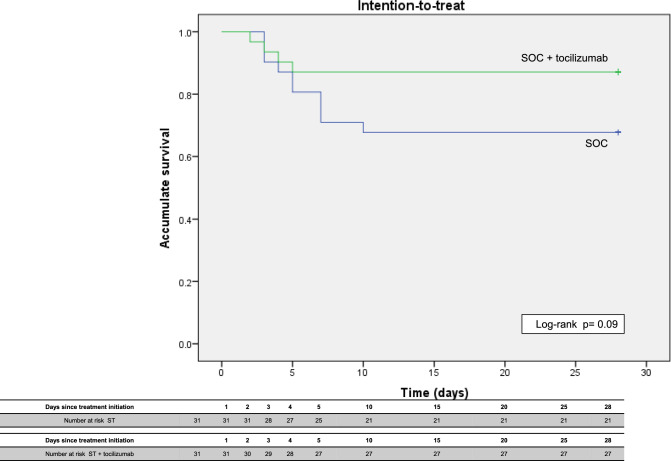
Cumulative survival by treatment group

The survival analysis showed a trend toward a higher accrued survival in the tocilizumab group, mainly after 10 days of randomization, however this difference did not reach statistical significance (Fig. 2).

### Safety outcomes

During the 28 days follow-up, 58 adverse events (AE) occurred in 27 participants (43.5%). Most of these AE were grade 1, with only 13% of the participants experiencing an AE grade 2 or 3 and none was a serious AE. The most frequently reported AE were liver enzymes alteration (14.5%), myopathy (14.5%) and delirium (11.3%). There were no differences between groups except for liver enzyme alterations, which was more frequently reported in the tocilizumab group (24.2% vs 3.4%, p = 0.021). All liver enzyme alterations recovered or improved during the follow up. Since the protocol consisted of a single dose of tocilizumab, no patients discontinued the medication due to an AE (Table 3).

**Table 3:** Safety analysis

	SOC group n = 29	Tocilizumab group n = 33	P value
Number of adverse events	28	30	
Participants with at least 1 adverse event	11 (37.9)	16 (48.5)	0.403
Median number of adverse events per participant [IQR]	0 [0–2]	0 [0–2]	1
Participants with adverse events grade 2 or 3	6 (20.7)	2 (6.1)	0.086
Blood system^1^	2 (6.9)	1 (3.0)	0.478
Cardiac^2^	2 (6.8)	2 (6.0)	0.898
Liver enzymes alteration^3^	1 (3.4)	8 (24.2)	**0.021**
Diarrhea	1 (3.4)	2 (6.1)	0.624
Delirium	4 (13.8)	3 (9.0)	0.554
Insomnia	0	1 (3.0)	0.351
Fungal infection^4^	0	1 (3.0)	0.351
Urinary Tract Infection	2 (6.9)	0	0.128
Bacterial pneumonia	2 (6.9)	0	0.128
Hyponatremia^5^	0	2 (6.1)	0.180
Muscle weakness	5 (17.2)	4 (12.1)	0.57

Significant P value (in bold)

^1^Blood system: Thrombocytopenia, Leucopenia, Thrombocytosis

^2^Cardiac: Chest pain, heart failure and atrial fibrillation

^3^Liver enzymes alteration: enzyme cytolysis elevation greater than 3 times the upper limit of normal or cholestasis enzymes greater than 2.5 times the upper limit of normal

^4^Fungal infection: a patient with a suspected pulmonary aspergillosis that was treated with voriconazole at the ICU

^5^Hyponatremia: plasma sodium < 130 mmol/L

Qualitative data is represented as total number and percentage

### Meta-analysis

**Fig. 3:**
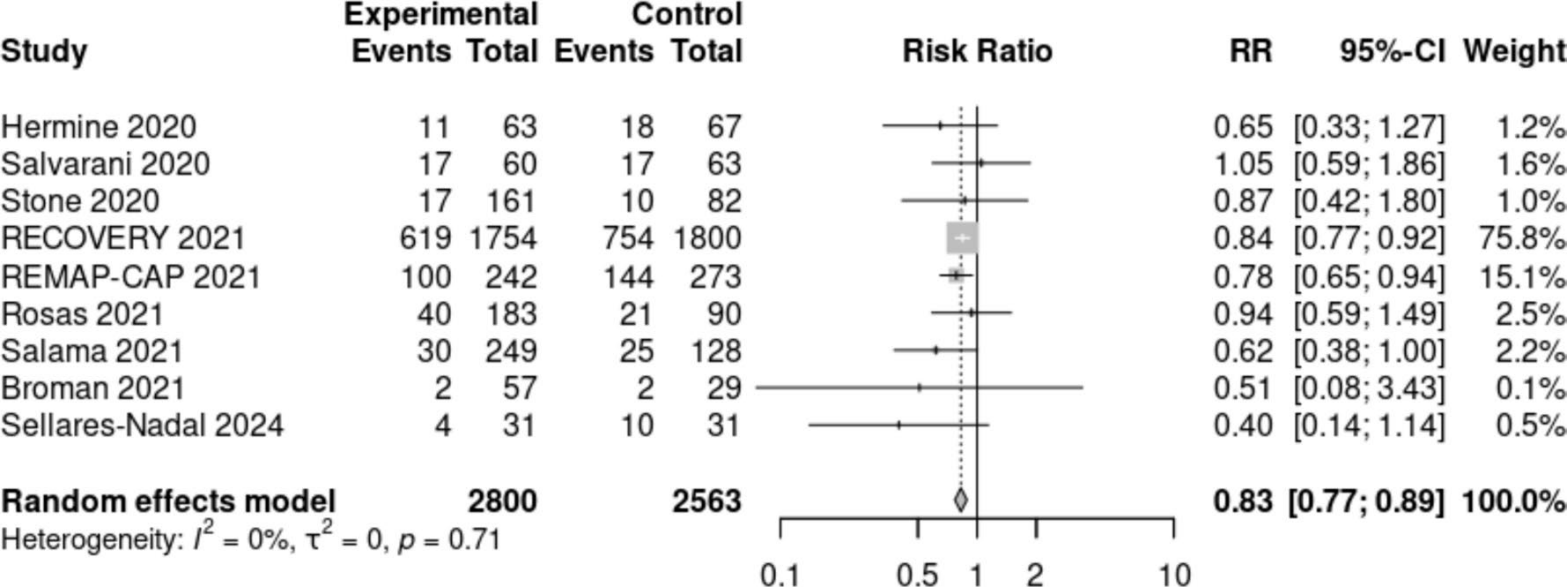
Efficacy forest plot of clinical trials assessing tocilizumab for COVID-19 pneumonia. The size of squares for risk ratio reflects weight of trial in pooled analysis. Horizontal bars represent 95% confidence intervals. *RR* Relative risk, *CI* confidence interval

We identified 2,262 citations in PubMed. After title and abstract screening and full text review, we included eight randomized clinical trials plus the present study in the meta-analysis (Figure S1 supplementary material) [8–11, 18–21]. All of them compared the use of tocilizumab versus standard care for COVID-19 pneumonia but some focused in critically ill patients and others in patients with severe disease. Only our study discriminated patients’ participation according to the IL-6 serum levels. The overall risk of bias was very low, with high quality across domains in all the studies included. Overall, 5277 participants were included; 2743 received tocilizumab and 2534 were controls. Pooled results showed that the RR for death or need of IMV was 0.83 (95% CI: 0.77–0.89) in participants receiving SOC plus tocilizumab, compared to participants receiving SOC (Fig. 3). Inter-study heterogeneity was very low.

## Discussion

In this randomized, controlled, open-label trial, we investigated the effect of tocilizumab on mortality and the need for IMV in adults with severe COVID-19 pneumonia and high IL-6 serum levels. Our primary analysis revealed a trend towards a lower rate of death or need for IMV in the tocilizumab group compared to the SOC group, although this difference was not statistically significant. It may be clinically significant, especially when considering that the trial was discontinued earlier, and the expected sample size was not reached, limiting the trial’s statistical power. Secondary outcomes showed similar trends favoring tocilizumab, including fewer days of IMV and shorter hospital stays. These results are novel since the study focusses on patients who might benefit the most from the IL-6 blockade strategy by selecting subjects with high IL-6 serum levels.

Our findings support previous studies that reported benefits of tocilizumab in reducing mortality and the need for mechanical ventilation. The RECOVERY trial [8] randomized 4116 hospitalized patients presenting hypoxia and systemic inflammation to receive tocilizumab versus SOC and found benefits in mortality and secondary outcomes. Similarly, in their study Salama et al. concluded that tocilizumab reduced the likehood of progression to mechanical ventilation or death [20]. The REMAP-CAP [9] focused in critically ill patients receiving organ support and assigned 865 subjects to receive an IL-6 antagonist or SOC. They found efficacy of IL-6 receptor antagonists in increasing the median of organ support-free days and in other secondary outcomes. Nevertheless, in none of these studies was it required to have elevated IL-6 to participate. The difference in the rate of the primary outcome was broader in our study than previous trials. Consistently, in the meta-analysis, the estimated RR for our study was half of the pooled estimate, driven by large trials such as RECOVERY and REMAP-CAP. This could be due to the selection of participants in whom the excessive inflammation and higher risk of progression were related to an increased IL-6. Consequently, this subpopulation could have a greater benefit from an IL-6 blockade strategy [9].

For instance, Stone et al. concluded that tocilizumab was not effective for preventing intubation or death in patients with moderate illness [11]. The difference with our results may be also explained by the fact that in their study, median IL-6 level was 24.4 pg/mL while in ours participants the median IL-6 serum levels was 76.4 pg/mL. A similar phenomenon occurs with the study by Salvarani et al., in which median IL-6 serum level in the participants was 42.1 pg/mL [19].

Interestingly, Wang et al*.* designed a clinical trial similar to ours, in which patients with high IL-6 serum levels were randomized to receive tocilizumab in addition to the standard of care. The primary outcome was clinical improvement, which was not statistically different in both groups. Despite the fact that this study was designed to select patients who might benefit most of an IL-6 blockade, IL-6 serum levels were only slightly above the normal limit (25 pg/mL), which might have diluted this effect [22].

Other trials not using personalized medicine driven by IL-6 serum levels were unable to demonstrate the benefit of tocilizumab. Rosas et al. concluded that the use of tocilizumab did not result in significantly better clinical status or lower mortality than placebo. Although in this trial, only 22% of the patients received glucocorticoids at inclusion in contraposition with our trial, in which 83% received concomitant steroids [10]. In their trial, Soin et al*.* did not find differences in illness progression in patients treated with tocilizumab but they included a highly heterogeneous sample population with patients presenting different ranges of severity [23].

Our study supports the safety and tolerability of tocilizumab, as no serious AEs were documented and only 6.1% of participants in the tocilizumab group presented a grade 2 or 3 AE. Notably, there were more transaminase elevations in the tocilizumab arm, but all were mild and transient, not requiring further intervention. This is consistent with the observed data reported in the package insert from the European Medicines Agency [24]. Moreover, there were no bacterial infections in These results enforce the statement that tocilizumab is useful in hospitalized patients with COVID-19. the tocilizumab group. Results from other clinical trials showed similar results, stressing the fact that the use of a limited number of doses in the context of a severe COVID-19 have a safety profile similar to other populations receiving tocilizumab.

The meta-analysis shows a lower proportion of death or IMV in patients treated with tocilizumab. The results are consistent among studies and the meta-analysis shows low heterogeneity due to the high quality of the trials included. Interestingly, 8 out of 9 clinical trials showed RR below one, indicating a protective effect of tocilizumab against death or IMV. Results in the trials administering tocilizumab regardless of the IL-6 inflammatory status showed a RR spinning around 0.8, while personalized medicine strategy based on IL-6 serum levels has a RR of 0.4 (95%CI 0.14–1.14) [25, 26].

The main strength of our study is the selection of a population at high risk of poor prognosis and with a highly likelihood of improving based on the tocilizumab mechanism of action. IL-6 serum level measurements can be easily implemented, and turnaround time can be less than few hours, allowing for the implementation of our personalized medicine strategy. Moreover, the randomized controlled design presents advantages by reducing biases and improving the reliability of the findings.

However, there are some limitations that need to be stated. First, the small sample size, that did not reach the calculated sample size, limits the interpretation of the results. In addition, patients were included during a two-year period, during which standard-care treatment and SARS-Cov2 variants changed, however this bias may have influenced both groups in the same proportion. On the other hand, our study included participants with infections from different strains over the time, and we did not find any differences regarding the time period in which participants were included in both efficacy and safety outcomes. Tocilizumab treatment may be equally effective regardless the circulating strain, and is unlikely that its efficacy could be compromised over the time. Finally, the last limitation is that during the study period, immunization was implemented, potentially affecting the outcomes and introducing variability in patient responses, but again patients were equally vaccinated in both groups.

Tocilizumab may reduce the risk of death or IMV in patients with severe COVID-19 pneumonia. This benefit could be higher among people with high IL-6 serum levels. A personalized treatment strategy driven by IL-6 serum levels in patients with severe COVID-19 pneumonia could maximize the benefits of tocilizumab and reduce the number of patients needed to treat to prevent a poor outcome, while optimizing the allocation of resources. Finally, tocilizumab in patients with severe COVID-19 pneumonia and high IL-6 serum levels is safe and well tolerated.

## Supplementary Information

Below is the link to the electronic supplementary material.

Supplementary file1 (DOCX 39 KB)

## Data Availability

The datasets generated and/or analyzed during the current study are available from the corresponding author on reasonable request. De-identified data will be shared after approval of a formal request. Data will be available for academic use only. Access may be restricted due to ethical considerations and patient confidentiality.
