# Supplementary material for: Efficacy of tocilizumab for hospitalized patients with COVID-19 pneumonia and high IL-6 levels: A randomized controlled trial
Source: Infection. 2025 Apr 15;53(5):1851–61. doi: 10.1007/s15010-025-02506-y (PMC12460590; doi:10.1007/s15010-025-02506-y)
Supplement: Supplementary file 1 — Supplementary file1 (DOCX 39 KB) [file 15010_2025_2506_MOESM1_ESM.docx]

**Table S1:** Baseline analytical parameters in both groups

|  | **SOC**  **n= 31 (%)** | **Tocilizumab**  **n= 31 (%)** |
| --- | --- | --- |
| **Baseline blood sample** |  |  |
| Lymphocytes (%) | 11.7 [9.5-17.5] | 11.2 [8.1-17.1] |
| D-dimer (ng/mL) | 238 [157-953] | 257 [181-439] |
| Fibrinogen (g/L) | 5.42 [4.9-6.32] | 5.78 [5.24-6.47] |
| Ferritin (ng/mL) | 580 [293-1022] | 765 [433-1083] |
| CRP (mg/dL) | 11.3 [7.56-17.7] | 15.3 [11.4-20] |

Data are expressed in median and [IQR]
SOC: Standard of care, CRP: C-reactive protein

**Table S2:** Efficacy outcomes in the modified intention-to-treat and modified-per-protocol analyses

| **Modified intention-to-treat analysis (mITT)** | **SOC**  **n= 29 (%)** | **Tocilizumab**  **n= 33 (%)** | **P value** |
| --- | --- | --- | --- |
| Death or IMV during the follow up | 8 (27.6) | 5 (15.2) | 0.230 |
| Death | 2 (6.9) | 1 (3.0) | 0.478 |
| IMV | 7 (24.1) | 5 (15.2) | 0.372 |
| **Modified per-protocol (mPP) analysis** | **SOC**  **n= 30 (%)** | **Tocilizumab**  **n= 31 (%)** | **P value** |
| Death or IMV during the follow up | 8 (26.7) | 4 (12.9) | 0.176 |
| Death | 1 (3.3) | 1 (3.2) | 0.982 |
| IMV | 8 (26.7) | 4 (12.9) | 0.176 |

IMV: Invasive mechanical ventilation, NIVM: Non-invasive mechanical ventilation, HFNC: High flow nasal cannula. SOC: standard of care.

The modified intention-to-treat analysis (mITT) reassigned the two patients that received tocilizumab despite being in the control group to the intervention group. The modified per-protocol analysis (mPP) considered these two patients failures in the control group and excluded the patient that did not receive tocilizumab despite being assigned to the intervention group.

**Table S3:** Secondary outcomes in the modified intention-to-treat and modified per-protocol analyses

| **Modified Intention-to-treat analysis (mITT)** | **SOC**  **n= 29 (%)** | **Tocilizumab**  **n= 33 (%)** | **P value** |
| --- | --- | --- | --- |
| ICU (IMV, NIVM, HFNC) | 12 (41.4) | 15 (45.5) | 0.747 |
| Death or hospitalized at the ICU at day 28 | 5 (17.2) | 3 (9.1) | 0.339 |
| Median days (IQR) under |  |  |  |
| Invasive mechanical ventilation | 14 (10-27) | 9 (3.5-27) | 0.343 |
| High-flow nasal cannula or NIMV | 3.5 (2-6.25) | 6 (2.75-7.75) | 0.235 |
| Length of hospital stay since the study inclusion, median, (IQR) | 8 (5.5-16) | 8 (4-13.5) | 0.461 |
| **Modified per protocol (mPP) analysis** | **SOC**  **n= 30 (%)** | **Tocilizumab**  **n= 31 (%)** | **P value** |
| ICU (IMV, NIVM, HFNC) | 14 (40) | 13 (41.9) | 0.881 |
| Death or hospitalized at the ICU at day 28 | 6 (20) | 2 (6.5) | 0.117 |
| Median days (IQR) under |  |  |  |
| Invasive mechanical ventilation | 19.5 (10.25-36.75) | 7.5 (2.25-12.75) | 0.073 |
| High-flow nasal cannula or NIMV | 3 (2-6) | 6 (3-6.75) | 0.211 |
| Length of hospital stay since the study inclusion, median, (IQR) | 8 (5.75-20) | 7 (4-13) | 0.132 |

IMV: Invasive mechanical ventilation, NIVM: Non-invasive mechanical ventilation, HFNC: High flow nasal cannula. SOC: standard of care group

**Figure S1- Flowchart of the studies included in the meta-analysis**

Randomized clinical trials assessed
 (n=14)

Final database search selection
 (n=8)

Manually included studies (n=1)

Full text assessed articles
 (n=28)

Records identified through PubMed searching between January 1, 2020 and May 31, 2024 (n=2262)

Records excluded after review of title or abstract (n=2234)

Non-randomized prospective studies (n= 14)

Lack of information regarding the main outcome of this meta-analysis (n=4)

No placebo controlled studies (n=2)

Studies included in this meta-analysis
 (n=9)
